# Development and Validation of Prognostic Nomograms for Periampullary Neuroendocrine Neoplasms: A SEER Database Analysis

**DOI:** 10.3390/curroncol30010028

**Published:** 2022-12-26

**Authors:** Jinghua Chen, Qichen Chen, Yiqiao Deng, Yujuan Jiang, Zhen Huang, Jianguo Zhou, Hong Zhao, Jianqiang Cai

**Affiliations:** 1 Department of Hepatobiliary Surgery, National Cancer Center/National Clinical Research Center for Cancer/Cancer Hospital, Chinese Academy of Medical Sciences and Peking Union Medical College, Beijing 100021, China; 2 Department of Colorectal Surgery, National Cancer Center/National Clinical Research Center for Cancer/Cancer Hospital, Chinese Academy of Medical Sciences and Peking Union Medical College, Beijing 100021, China

**Keywords:** periampullary, neuroendocrine neoplasms, nomogram, prognosis, SEER

## Abstract

(1) Background: Periampullary neuroendocrine neoplasms (NENs) are rare tumors that lack a prognostic prediction model. We aimed to design comprehensive and effective nomograms to predict prognosis; (2) Methods: Univariate and multivariate Cox analyses were used to screen out significant variables for the construction of the nomograms. The discrimination and calibration of the nomograms were carried out using calibration plots, concordance indices (C-indices), and area under time-dependent receiver operating characteristic curves (time-dependent AUCs). Decision curve analysis (DCA) was used to compare the clinical applicability of the nomograms, TNM (Tumor- Node-Metastasis) stage, and SEER stage; (3) Results: The independent risk factors for overall survival (OS) and cancer-specific survival (CSS) of patients with periampullary NENs included age, tumor size, histology, differentiation, N stage, M stage, and surgery, which were used to construct the nomograms. The calibration curves and C-indices showed a high degree of agreement between the predicted and actual observed survival rates. The AUCs displayed good calibration and acceptable discrimination of the nomograms. Additionally, the DCA curves indicated that the nomograms showed better clinical applicability; (4) Conclusions: We developed and validated nomogram prognostic models for patients with periampullary NENs. The nomograms provided insightful and applicable tools to evaluate prognosis.

## 1. Introduction

Periampullary tumors arise within 2 cm of the ampulla of the duodenum and include ampullary tumors, pancreatic head cancer, lower common bile duct cancer, ampullary cancer, and periampullary duodenal cancer [1]. Periampullary neuroendocrine neoplasms (NENs) are extremely rare tumors, accounting for less than 1% of all gastrointestinal neuroendocrine tumors and less than 2% of all tumors of the ampullary region [2]. Periampullary NENs are heterogeneous tumors that originate from the neuroendocrine cells of the gastrointestinal tract or pancreas [3,4]. According to the updated World Health Organization (WHO) classification, NENs include low-grade neuroendocrine tumors (NETs) and high-grade neuroendocrine carcinomas (NECs) [5]. The prevalence of periampullary NENs has been consistently increasing, which may be attributable to improvements in the way NENs are diagnosed, including better imaging tests and endoscopy, and increased awareness of these tumors [3,6].

The complexity of the periampullary anatomy makes it difficult to determine the origin of these tumors [7]. Fortunately, advances in techniques have helped with diagnosis as well as with defining the extent of the lesion and potential resectability. For periampullary tumors, surgery is the recommended treatment, and pancreaticoduodenectomy (PD) is the classic procedure. Endoscopic removal is being extended to different lesions with encouraging preliminary results [8,9]. At present, there are very few studies on periampullary NENs. A retrospective study of 101 patients with ampullary and duodenal NETs demonstrated lymph nodal involvement to be common among those >1 cm in size, and resection with lymphadenectomy for these larger tumors is recommended [10]. Another National Cancer Database (NCDB)-based study compared patients with ampullary, duodenal, or pancreatic head NETs; this study was, however, limited by unknown data [11]. Nevertheless, there is no individual prediction model to evaluate the prognosis of patients with periampullary NENs. The American Joint Committee on Cancer (AJCC) staging system is the most frequently used system to evaluate prognosis in patients with NENs. It can be inferred from research on NETs involving other sites that the major limitations of the AJCC stage include low accuracy, disregard of other factors, and poor performance in predicting individual survival risk [12,13].

Given the rarity of periampullary NENs, and in particular NENs of the ampulla, there is a clear gap in knowledge in the current literature, which is limited to primarily small case series. In the present study, using the dataset from the Surveillance, Epidemiology and End Results (SEER) database, we aimed to identify prognostic factors for patients with periampullary NENs, and then to develop and validate nomogram prognostic models to visually predict the overall survival (OS) and cancer-specific survival (CSS) in patients with periampullary NENs.

## 2. Materials and Methods

### 2.1. Data Resource and Patients

The data were retrieved from the SEER database based on the November 2020 submission, derived from 18 cancer registries across the United States of America (U.S.A), covering approximately 30% of incident cases of the whole country [14]. We used the SEER*Stat software (version 8.3.9) to identify patients diagnosed with NENs located in the periampullary region between 2010 and 2015 in the SEER database as follows: International Classification of Diseases for Oncology (ICD-O-3) codes: 8013 (large cell neuroendocrine carcinoma), 8240 (carcinoid not otherwise specified), 8246 (neuroendocrine not otherwise specified), and 8249 (atypical carcinoid) and primary site codes: C17.0 (duodenum), C24.0 (extrahepatic bile duct), C24.1 (Ampulla of Vater) and C25.0 (head of pancreas).

**Figure 1:**
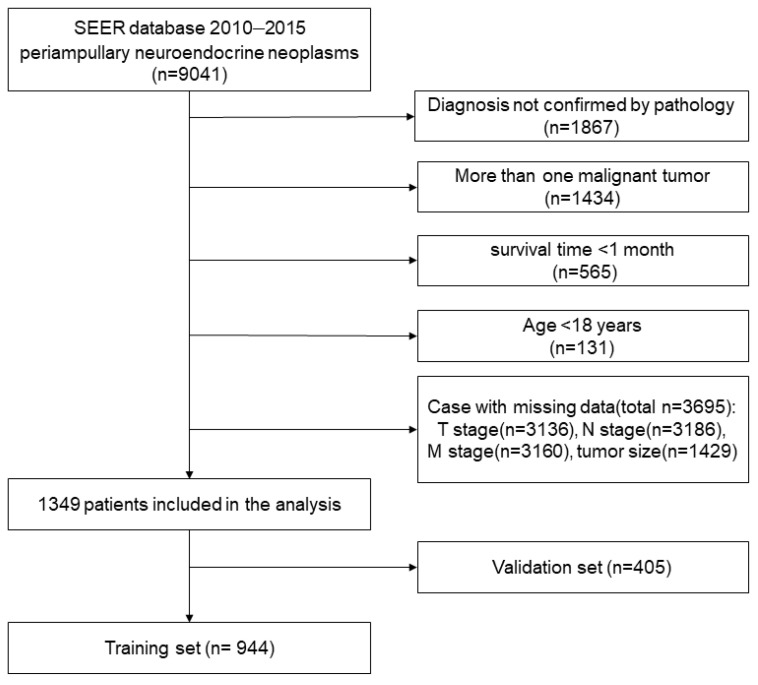
Flow diagram for the selection of periampullary neuroendocrine neoplasms included in the final analyses based on the SEER set.

Patients were enrolled in this study based on the following criteria: (1) having been diagnosed with periampullary NENs; (2) having demographic variables, including age, sex, race, geographic region, and income, available; and (3) having clinicopathological information, including primary tumor site, grade, histological type, TNM (Tumor-Node-Metastasis) stage, tumor size, and surgical information, available. The exclusion criteria were as follows: (1) having more than one malignant tumor; (2) having a survival time <1 month; and (3) being <18 years of age. Ultimately, 1349 eligible patients were selected for this cohort. A flow diagram of the selection process is presented in Figure 1. This study was exempt from the Ethics Review Committee of the Cancer Hospital, Chinese Academy of Medical Sciences. No personal identifying information was used in the study. Therefore, informed consent was not required.

### 2.2. Variable Assessment and Statistical Analysis

Demographic and clinical information were extracted from the SEER database. The variables included age at diagnosis (<60 or ≥60 years), sex, race (white, black, or other), tumor characteristics (histology, grade, tumor-node-metastasis stage), tumor size, median household income (<60,000 USD, 60,000–69,999 USD, or >70,000 USD), geographic region (metropolitan areas or nonmetropolitan), surgery, lymph node examination (LNE), survival time, and cause of death. Tumor size was divided into two groups according to the optimal cut-off value (2.7 cm) for survival obtained by X-title analysis. OS was defined from the date of diagnosis to the date of death due to any cause. CSS was defined from the date of diagnosis to the date of death due to lung carcinoid tumors.

For predicting the risk factors for survival in patients with periampullary NENs, patients who were ultimately enrolled in this study were randomly divided into two subgroups, defined as the training set and the validation set (7:3). Categorical variables were reported as the number and percentage and compared using χ^2^ tests. X-tile analysis was conducted to determine the optimal segmentation threshold for continuous measurements. Survival analyses were estimated by the Kaplan–Meier analysis and log-rank test. Potential risk variables (*p* < 0.1 in univariate Cox regression) were entered into multivariate Cox proportional hazard models. Model in Enter manner was applied to estimate the hazard ratio (HR), and the corresponding 95% confidence interval (CI) was also calculated for every potential prognostic variable. The above analyses were performed in SPSS 25.0 (IBM Corp, Armonk, NY, USA).

Based on the results of the multivariate analysis, nomograms were formulated with risk factors (*p* < 0.05) by using R version 3.5.1 (http://www.r-project.org/, accessed on 3 October 2021). The comparison between the nomogram-predicted and actual outcomes were conducted by calibration plots. Concordance indices (C-indices) were used for comparing the nomograms to the performance predicted results. Receiver operating characteristic (ROC) curves were used to determine the sensitivity and specificity of the nomograms. Furthermore, a decision curve analysis (DCA) was used for the threshold probability range of the nomograms in association with the TNM staging and SEER staging systems. In addition, the nomograms were also compared to the TNM stage and SEER stage in terms of AUC and C-indices. Differences with *p* < 0.05 (two-sided) were considered statistically significant.

## 3. Results

### 3.1. Demographic and Clinical Characteristics

A total of 1349 patients with periampullary NEN were selected in this process, of which 944 patients were randomly assigned to the training set and 405 patients were assigned to the validation set. Due to missing data, such as stage, we excluded distal bile duct NENs, and, ultimately, included only duodenal NENs (d-NENs), ampullary NENs (a-NENs), and pancreatic head NENs (p-NENs). The median follow-up was 52 [interquartile range (IQR): 37–71] months for the entire population; 51 (IQR: 36–70) months in the training cohort; and 50 (IQR: 44–73) months for the validation cohort. All demographic and clinical characteristics of these patients with periampullary NENs are summarized in Table 1. The training and validation cohorts were comparable in terms of the demographic and clinical characteristics (all *p* > 0.05).

### 3.2. Independent Predictors in the Training Set and Survival Outcomes

**Figure 2:**
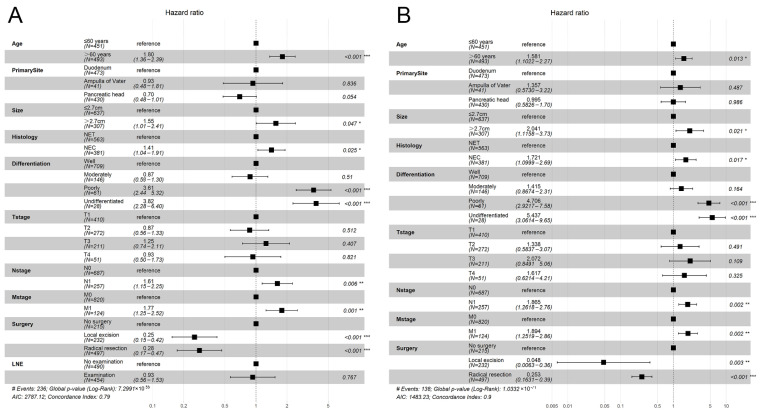
Forest plot for multivariate Cox regression analysis. (**A**) Forest plot for overall survival; (**B**) Forest plot for cancer–specific survival. (* *p* < 0.05; ** *p* < 0.01; *** *p* < 0.0001).

The HRs for OS according to all variables in the univariate and in the multivariate Cox proportional hazards models are shown in Table S1 and Figure 2, respectively. In the univariate analysis, we found that age > 60 years (*p* < 0.001), primary site (*p* < 0.001), tumor size (*p* < 0.001), histology (*p* < 0.001), differentiation (*p* < 0.001), T stage (*p* < 0.001), N stage (*p* < 0.001), M stage (*p* < 0.001), surgery (*p* < 0.001) and LNE (*p* = 0.001) were identified as significant prognostic factors for OS (Table S1). When those variables were further analyzed in the multivariate analysis, we found that age (*p* < 0.001), tumor size > 2.7 cm (*p* = 0.047), NEC (*p* = 0.025), poorly differentiated (*p* < 0.001), undifferentiated (*p* < 0.001), N1 stage (*p* = 0.006), M1 stage (*p* = 0.001), local excision (*p* < 0.001), and radical resection (*p* < 0.001) remained statistically significant, indicating that they are significant, independent predictors for OS (Figure 2A).

The HRs for CSS according to all variables in the univariate and multivariate Cox proportional hazards models are shown in Table S1 and Figure 2, respectively. In the univariate analysis, we found that age > 60 years (*p* = 0.005), primary site (*p* < 0.001), tumor size (*p* < 0.001), histology (*p* < 0.001), differentiation (*p* < 0.001), T stage (*p* < 0.001), N stage (*p* < 0.001), M stage (*p* < 0.001) and surgery (*p* < 0.001) were identified as significant prognostic factors for CSS (Table S1). When those variables were further analyzed in the multivariate analysis, we found that age (*p* = 0.013), tumor size > 2.7 cm (*p* = 0.021), NEC (*p* = 0.017), poorly differentiated (*p* < 0.001), undifferentiated (*p* < 0.001), N1 stage (*p* = 0.002), M1 stage (*p* = 0.002), local excision (*p* = 0.003), and radical resection (*p* < 0.001) remained statistically significant, indicating that they are significant, independent predictors for CSS (Figure 2B).

The 1-, 3-, and 5-year OS rates of the training set were 89.4%, 83.1%, and 77.5%, respectively. The 1-, 3-, and 5-year CSS rates of the training set were 92.8%, 88.3%, and 86.0%, respectively. The 1-, 3-, and 5-year OS rates of the validation set were 93.4%, 85.9%, and 81.7%, respectively. The 1-, 3-, and 5-year CSS rates of the validation set were 94.8%, 91.1%, and 88.4%, respectively. Subgroup survival analyses of independent prognostic factors identified above, including age, tumor size, histology, differentiation, N stage, M stage, and surgery, were conducted based on Kaplan–Meier analysis with the log-rank test in the training set. The associations between several important predictors and OS or CSS are further illustrated in Figures S1–S4. We also found that the survival benefit of patients who underwent local excision was better than that of patients who underwent radical resection (Figures S2D and S4D).

### 3.3. Nomogram Development and Validation

**Figure 3:**
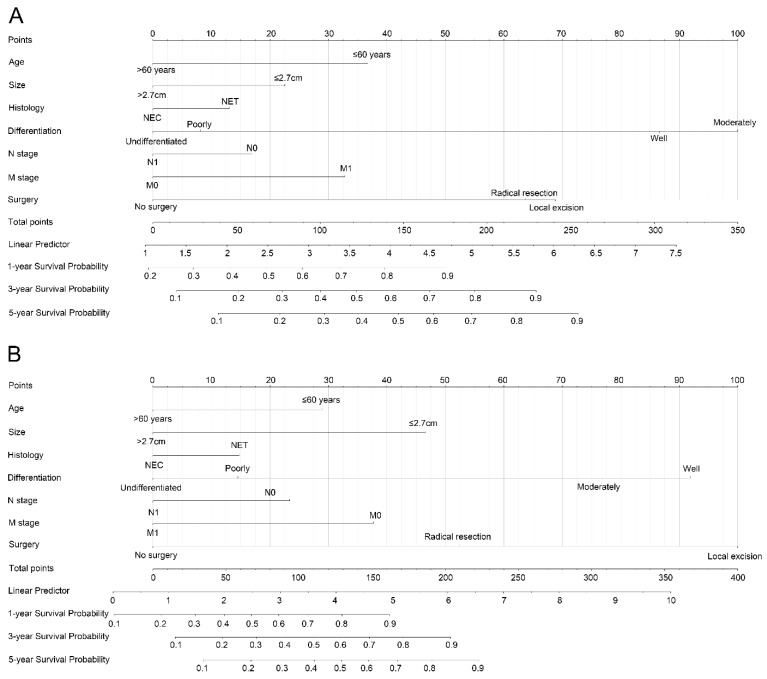
Prognostic nomogram predicting the probability of survival. (**A**) Nomogram for OS; (**B**) Nomogram for CSS. The total scores for each variable present the probabilities of 1-, 3- and 5-years OS or CSS.

Nomograms were constructed based on the risk factors of the Cox proportional hazards regression in the training cohort predicting 1-, 3-, and 5-year OS and CSS (Figure 3). Age, tumor size, histology, differentiation, N stage, M stage, and surgery were included in the predict models. Each subtype within these above variables was assigned a score on the point scale. The 1-, 3-, and 5-year survival probability could be easily calculated by adding because each patient had a different overall score.

**Figure 4:**
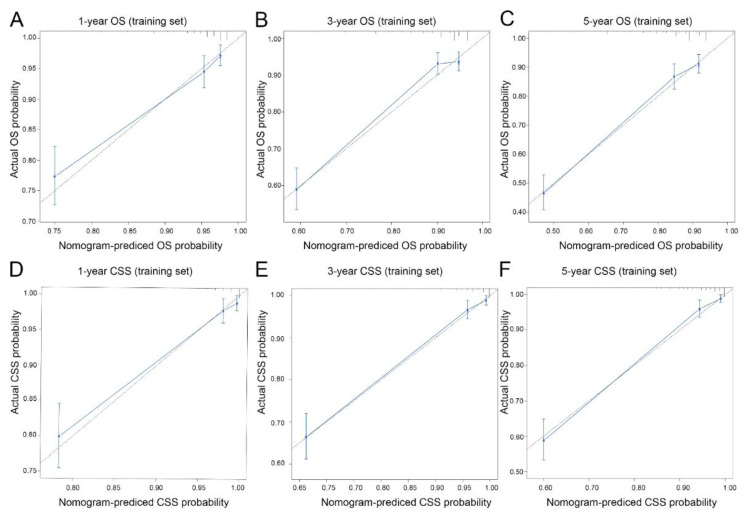
Calibration curves for predicting overall survival and cancer-specific survival in the training set. Overall survival (**A**–**C**): 1-year overall survival (**A**), 3-year overall survival (**B**) and 5-year overall survival (**C**). Cancer-specific survival (**D**–**F**): 1-year cancer-specific survival (**D**), 3-year cancer-specific survival (**E**) and 5-year cancer-specific survival (**F**).

**Figure 5:**
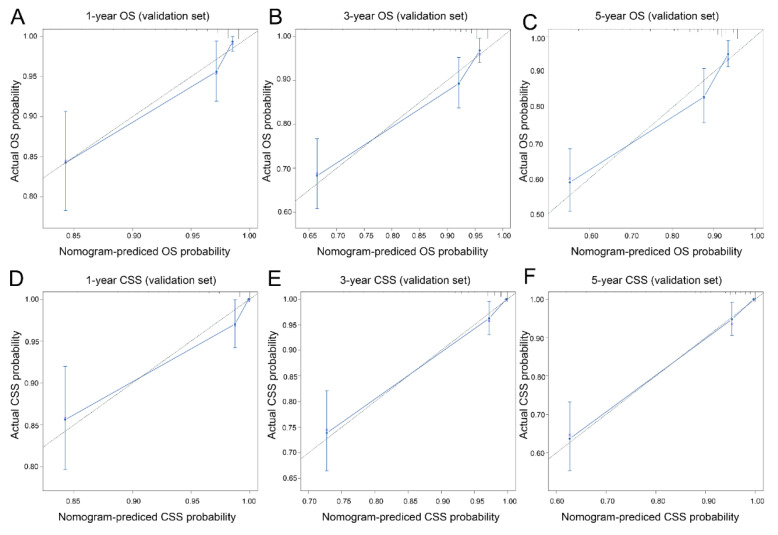
Calibration curves for predicting overall survival and cancer-specific survival in the validation set. Overall survival (**A**–**C**): 1-year overall survival (**A**), 3-year overall survival (**B**) and 5-year overall survival (**C**). Cancer-specific survival (**D**–**F**): 1-year cancer-specific survival (**D**), 3-year cancer-specific survival (**E**) and 5-year cancer-specific survival (**F**).

**Figure 6:**
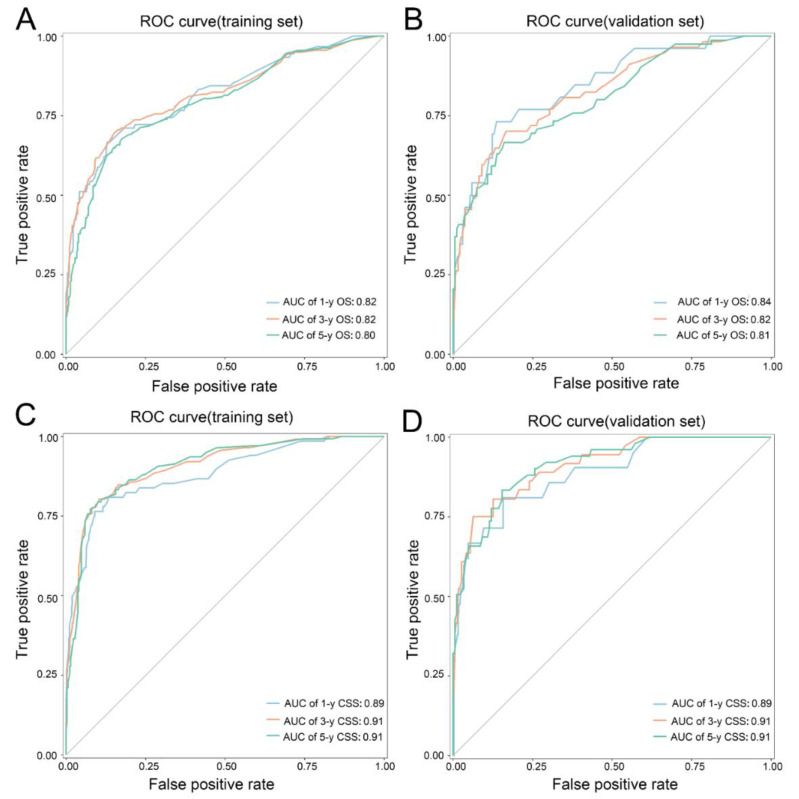
Receiver operating characteristic curves of the nomograms for survival in both the training and validation sets. Training set (**A**,**C**): Overall survival (**A**) and cancer-specific survival (**C**). Validation set (**B**,**D**): Overall survival (**B**) and cancer-specific survival (**D**).

**Figure 7:**
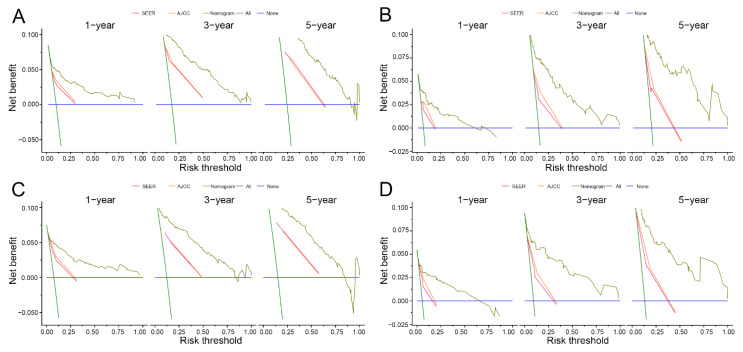
Decision curve analysis of the nomograms for survival in both the training and validation sets. Training set (**A**,**C**): Overall survival (**A**) and cancer−specific survival (**C**). Validation set (**B**,**D**): Overall survival (**B**) and cancer—specific survival (**D**).

Calibration curves of 1-year, 3-year and 5-year OS and CSS in the training dataset and validation dataset were established. The results exhibited good calibration between the nomogram predictions and actual observations (Figure 4 and Figure 5, respectively). In addition, better AUCs were found both in the training and validation sets (Figure 6). In both OS and CSS, the AUCs of the nomograms were significantly higher than those of the SEER stage and AJCC stage, indicating that the nomograms had better discrimination (Table 2). In the training set, the C-indexes for the OS and CSS nomograms were 0.888 (95% CI: 0.860–0.917) and 0.890 (95% CI: 0.872–0.925), while the C-indexes were 0.881 (95% CI: 0.836–0.925) and 0.897 (95% CI: 0.856–0.938) in the validation set. In particular, in both the training and validation sets, the OS nomogram and CSS nomogram displayed significantly better performances than the TNM stage (Table 3). The DCA results showed that the nomograms showed similar clinical applicability to that of the TNM stage and SEER stage (Figure 7).

## 4. Discussions

Periampullary NENs are relatively rare tumors, with little clinical evidence regarding prognosis. This study constructed and validated prognostic nomogram models for both the OS and CSS of periampullary NENs based on the public database SEER. By both internal and external validation, the nomograms used displayed comparable outcomes to those of the TNM stage and SEER stage. Seven variables were selected by Cox regression and incorporated into the nomogram. Measured by standard deviation along with nomogram scales, the degree of differentiation and surgery were the most important factors, with the remaining factors being age, tumor size, pathological type, N stage, and M stage. This was the first study to establish prognostic models for patients with periampullary NENs. The prognostic nomograms could facilitate clinical prognostic evaluation and personalized treatment.

In predicting prognosis, we treated the NENs at all three sites as a whole. Whether or not the site of tumor origin (ampullary, duodenum, or pancreatic head) determines the prognosis has always been a concern. Previous reports have suggested that tumors arising at the ampulla of Vater are associated with a larger size, higher grade, and increased risk of nodal metastases [15,16]. Both prior single-institution case series [17] and our findings show a higher rate of lymph node metastasis in ampullary NENs at diagnosis (ampullary vs. duodenum vs. pancreatic head: 54.4% vs. 15.0% vs. 37.0%, respectively, *p* < 0.001). Nevertheless, similar oncologic outcomes were obtained by Schmocker RK and his colleagues despite more aggressive histopathologic features, suggesting that the disease biology of d-NENs may be more indolent than that of a-NENs or p-NENs [18]. In agreement with Schmocker RK et al., we also found no significant difference for the prognosis of NENs at different primary sites, which indicates that the tissue of origin does not appear to impact long-term outcomes. Other researchers have suggested the same [15,18]. Another possible reason is that a large part of the data in the SEER database has been lost, resulting in a smaller number of cases of NENs at the ampulla of Vater. Moreover, neuroendocrine tumors, especially pancreatic NENs, are difficult to detect in the early stages due to insidious onset, and are detected in most patients at an advanced stage (patients in stage IV: duodenum 3.7% vs. pancreatic head 22.9%, *p* < 0.001).

While most subtypes are afforded independent staging systems in the latest AJCC staging system, ampullary and duodenal NENs remain combined into one group. This is in part due to anatomic proximity, and determination of the true tissue of origin at this location can be challenging. Although the head of the pancreas is a part of the pancreas, it is also close to the ampulla and the distal bile duct, and the clinical symptoms, surgical methods, and prognosis are similar to those for the duodenum and ampulla. A series of studies has shown that, although the biological behaviors of the three sites are different, this did not affect patient survival [18], which is consistent with the results of study. Therefore, we undertook to evaluate their roles as independent prognostic factors and to build a prognostic model to predict survival by combining the three factors.

Our results show that only the N stage and M stage have predictive effects on patient prognosis, whereas the T stage has no significant effect on the CSS or OS of patients. Distant metastasis measured by M stage is a recognized risk factor [11,15], while T stage and N stage have always had conflicting results. T stage is based on tumor size and depth of invasion. Tumor size has been regarded as a prognostic marker for adenocarcinoma of the periampullary region [10], and a series of studies has proven that tumor size is closely related to lymph node metastasis and prognosis in gastrointestinal NENs [19,20]. For d-NENs, tumor size is an important factor in determining the surgical approach [21,22,23]. However, studies have shown differing results. Although current studies on periampullary NENs are very limited, several small, retrospective studies have also found tumor size not to be associated with tumor recurrence and survival [4,15]. Whether the depth of tumor invasion is an independent prognostic factor is currently uncertain. According to the N stage, some have suggested that the presence of lymph node involvement contributes to a worse prognosis [24], while numerous others have shown that lymph node metastases do not appear to impact survival [10,16,21]. These are topics that clearly require additional study. To a certain extent, this suggests that the TNM stage may not be the most accurate tool for prognostic prediction and that the prognosis for patients at the same stage may be heterogeneous. Therefore, we urgently need to establish a prediction model to predict prognosis more accurately for patients with periampullary NENs.

The choice of surgical procedure has also been considered a possible reason for the same prognosis in patients despite different tissues of origin. In our cohort, patients with a-NENs and p-NENs were more likely to choose radical surgery, while patients with d-NENs were more inclined to choose local or endoscopic excision. However, previous studies have found a high rate of lymph node metastasis in d-NENs; even given a tumor ≤ 1 cm, there is still a 40% incidence of lymph node metastasis (LNM) [10]. Studies regarding endoscopic or surgical therapy for ampullary tumors are heterogeneous. There is still no definitive criterion for the choice of endoscopic or surgical resection. Gay-Chevallier S et al. [25] suggested that a less invasive therapeutic strategy appeared more suitable than oncological surgery for nonmetastatic d-NENs. A small-scale comparative study conducted by Lee SW and his colleagues found that the pathologic complete response rate of lesions ≥ 11 mm in the surgical treatment group was higher than that in the endoscopic treatment group [26]. In addition, surgical treatment was mostly beneficial among patients with p-NENs > 2 cm, a Ki-67 index ≥ 3%, and lesions located at the pancreatic head, as identification of the LNM was most common among individuals with these tumor characteristics [27]. In contrast, data on surgical ampullectomies are very few, whether adenocarcinoma or NENs are present [28]. Research by Beger HG et al. [29] showed extended resection for low-risk periampullary cancer to be associated with a significant increase in procedure-related biliary and duodenal complications. In summary, the therapeutic value of radical resection remains controversial among patients undergoing surgery for periampullary NENs, despite the possibility that removal of the LNM may decrease locoregional recurrence. Additionally, consensus guidelines and national and international recommendations are lacking. Our nomograms show that, compared with radical resection, the corresponding score of local excision was lower and the survival rate was better. LNE also did not confer survival benefits to patients. The possible reason for this is that radical resection increases the occurrence of postoperative complications. The optimal surgical approach requires further exploration and has implications for individual treatment.

In addition, other factors, including age, tumor differentiation, and histology, were associated with recurrence and/or survival [11,30,31,32]. The current study proved that tumor differentiation and histology were important prognostic factors for CSS and OS. Older age was significantly related to worse survival. Similar to our results, a previous study found that patients with NETs were younger than those with NECs [33].

There are several limitations to the present study. First, as a retrospective study, there exist inherent biases. The large percentage of missing data might have introduced some selection bias. Second, some important variables, such as the Ki-67 index and mitotic index, were unable to be obtained from the SEER database. The Ki-67 index and mitotic index could be used to access the aggressiveness of NETs, which have been endorsed by the European Neuroendocrine Tumor Society (ENETS) grading system [5,34]. Third, postoperative complications cannot be assessed in the SEER database. Large prospective studies should be conducted to verify our nomograms in future. Despite these limitations, our prognostic nomograms are important and effective models for providing an accurate and individualized survival prediction in patients with periampullary NENs.

## 5. Conclusions

We developed and validated nomogram prognostic models for periampullary NET patients. The proposed nomograms show better prognostic performance and clinical applicability, similar to those of the TNM stage and SEER stage. Researchers, clinicians, and patients can easily predict the survival probability for each individual patient using nomograms. In addition, we should pay more attention to patients with poor prognostic factors (age > 60 years, tumor > 2.7 cm, NEC, worse differentiation, N1 stage, M1 stage, and no surgery), and we recommend more active treatment and closer follow-up. In the future, external verifications of the prediction models are needed to prove their good prediction ability, and the universality of these models should be confirmed through prospective or multi-center studies.

## Figures and Tables

**Table 1:** Demographic and clinical characteristics of patients with periampullary neoplasms.

Characteristic	Whole Population [n = 1349 (%)]	Training Cohort [n = 944 (%)]	Validation Cohort [n = 405 (%)]	*p*-Value
Age				
≤60 years	659 (48.9)	451 (47.8)	208 (51.4)	0.228
>60 years	690 (51.1)	493 (52.2)	197 (48.6)	
Sex				
Female	678 (50.3)	465 (49.3)	213 (52.6)	0.262
Male	671 (49.7)	479 (50.7)	192 (47.4)	
Race				
White	954 (70.7)	674 (71.4)	280 (69.1)	0.684
Black	253 (18.8)	174 (18.4)	79 (19.5)	
Other	142 (10.5)	96 (10.2)	46 (11.4)	
Geographic region				
Rural/urban	142 (10.5)	90 (120.5)	43 (10.5)	0.943
Metropolitan	1207 (89.5)	845 (89.5)	362 (89.5)	
Income (USD)				
≤60,000	484 (35.9)	333 (35.3)	151 (37.3)	0.377
60,000–70,000	404 (29.9)	304 (32.2)	100 (24.7)	
>70,000	461 (34.2)	307 (32.5)	154 (38.0)	
Primary site				
Duodenum	667 (49.4)	473 (50.1)	194 (47.9)	0.672
Ampulla	57 (4.2)	41 (4.3)	16 (4.0)	
Pancreatic head	625 (46.2)	430 (45.6)	195 (48.1)	
Tumor size				
≤2.7 cm	907 (67.2)	637 (67.5)	270 (66.7)	0.771
>2.7 cm	442 (32.8)	307 (32.5)	135 (33.3)	
Histology				
Neuroendocrine tumors	790 (58.6)	563 (59.6)	227 (56.0)	0.220
Neuroendocrine carcinomas	559 (41.4)	381 (40.4)	178 (44.0)	
Differentiation				
Well differentiated	1012 (75.0)	709 (75.1)	303 (74.8)	0.677
Moderately differentiated	215 (15.9)	146 (15.5)	69 (17.0)	
Poorly differentiated	86 (6.4)	61 (6.4)	25 (6.2)	
Undifferentiated	36 (2.7)	28 (3.0)	8 (3.0)	
T stage				
T1	586 (43.4)	410 (43.4)	176 (43.4)	0.819
T2	393 (29.2)	272 (28.8)	121 (29.9)	
T3	302 (22.4)	211 (22.4)	91 (22.5)	
T4	68 (5.0)	51 (5.4)	17 (4.2)	
N stage				
N0	987 (73.2)	687 (72.8)	300 (74.1)	0.622
N1	362 (26.8)	257 (27.2)	105 (25.9)	
M stage				
M0	1175 (87.1)	820 (86.9)	355 (87.7)	0.692
M1	174 (12.9)	124 (13.1)	50 (12.3)	
TNM stage				
I	655 (48.6)	450 (47.7)	205 (50.6)	0.644
II	382 (28.3)	268 (28.4)	114 (28.1)	
III	138 (10.2)	102 (10.7)	36 (8.9)	
IV	174 (12.9)	124 (13.2)	50 (12.4)	
SEER stage				
Localized	809 (60.0)	565 (59.9)	244 (60.2)	0.772
Regional	357 (26.5)	247 (26.2)	110 (27.2)	
Distant	183 (13.5)	132 (13.9)	51 (12.6)	
Surgery				
No surgery	279 (20.7)	195 (20.7)	84 (20.7)	0.889
Local excision	348 (25.8)	252 (26.7)	94 (23.7)	
Radical resection	722 (53.5)	497 (52.6)	225 (55.6)	
Lymph node examination				
No	704 (52.2)	490 (51.9)	214 (52.8)	0.753
Yes	645 (47.8)	454 (48.1)	191 (47.2)	

Abbreviation: TNM stage: Tumor-Node-Metastasis stage; SEER: Surveillance, epidemiology and end results.

**Table 2:** Comparison of the AUC of the SEER stage, TNM stage and Nomogram.

Survival Types		Tumor Stage Types	Training Set			Validation Set		
			AUC	95% CI	*p*	AUC	95% CI	*p*
OS	1-year	Nomogram	0.817	0.764–0.869		0.845	0.763–0.927	
		SEER stage	0.716	0.659–0.773	<0.001	0.740	0.651–0.829	0.019
		TNM stage 7th	0.686	0.625–0.746	<0.001	0.720	0.629–0.829	0.023
	3-year	Nomogram	0.817	0.776–0.859		0.825	0.763–0.887	
		SEER stage	0.728	0.684–0.772	<0.001	0.690	0.627–0.763	<0.001
		TNM stage 7th	0.713	0.667–0.760	<0.001	0.661	0.554–0.705	<0.001
	5-year	Nomogram	0.796	0.756–0.836		0.806	0.744–0.868	
		SEER stage	0.683	0.639–0.727	<0.001	0.651	0.580–0.723	<0.001
		TNM stage 7th	0.667	0.631–0.723	<0.001			<0.001
CSS	1-year	Nomogram	0.889	0.843–0.934		0.885	0.808–0.962	
		SEER stage	0.805	0.754–0.857	<0.001	0.790	0.711–0.869	0.004
		TNM stage 7th	0.779	0.724–0.833	<0.001	0.749	0.660–0.838	<0.001
	3-year	Nomogram	0.913	0.883–0.943		0.911	0.861–0.961	
		SEER stage	0.834	0.796–0.873	<0.001	0.795	0.724–0.866	<0.001
		TNM stage 7th	0.819	0.778–0.859	<0.001	0.764	0.690–0.838	<0.001
	5-year	Nomogram	0.911	0.882–0.940		0.913	0.870–0.956	
		SEER stage	0.838	0.801–0.876	<0.001	0.753	0.679–0.828	<0.001
		TNM stage 7th	0.828	0.788–0.868	<0.001	0.731	0.653–0.810	<0.001

Abbreviation: AUC: Area under time-dependent receiver operating characteristic curves; SEER: Surveillance, epidemiology and end results; TNM stage: Tumor-Node-Metastasis stage; OS: Overall survival; CSS: Cancer-specific survival.

**Table 3:** Comparison of C-indexes of SEER stage, TNM stage and Nomogram.

Survival Types	Tumor Stage Types	Training Set			Validation Set		
		C-Index	95% CI	*p*	C-Index	95% CI	*p*
OS	Nomogram	0.888	0.860–0.917		0.881	0.836–0.925	
	SEER stage	0.906	0.876–0.933	0.882	0.861	0.805–0.918	0.226
	TNM stage 7th	0.868	0.834–0.902	0.097	0.814	0.749–0.878	0.016
CSS	Nomogram	0.890	0.872–0.925		0.897	0.856–0.938	
	SEER stage	0.904	0.876–0.933	0.695	0.861	0.805–0.918	0.063
	TNM stage 7th	0.868	0.834–0.902	0.014	0.814	0.749–0.879	0.002

Abbreviation: SEER: Surveillance, epidemiology and end results; TNM stage: Tumor-Node-Metastasis stage; OS: Overall survival; CSS: Cancer-specific survival.

## Data Availability

These data were derived from the SEER cancer database available in the public domain: https://seer.cancer.gov (accessed on 8 June 2022). The data that support the findings of this study are available from the corresponding author upon reasonable request.

## Supplementary Materials

The following supporting information can be downloaded at: https://www.mdpi.com/article/10.3390/curroncol30010028/s1. The supplementary materials have been uploaded to the submission system. Figure S1: Overall survival analysis in the training set. (A) age ≤ 60 years vs. age > 60 years, (B) tumor size ≤ 2.7 cm vs. tumor size > 2.7 cm, (C) neuroendocrine tumors vs. neuroendocrine carcinoma; Figure S2: Overall survival analysis in the training set. (A) well differentiated vs. moderately differentiated vs. poorly differentiated vs. undifferentiated; (B) N0 stage vs. N1 stage; (C) M0 stage vs. M1 stage; (D) No surgery vs. local excision vs. radical resection; Figure S3: Cancer-specific survival analysis in the training set. (A) age ≤ 60 years vs. age > 60 years, (B) tumor size ≤ 2.7 cm vs. tumor size > 2.7 cm, (C) neuroendocrine tumors vs. neuroendocrine carcinoma; Figure S4: Cancer-specific survival analysis in the training set. (A) well differentiated vs. moderately differentiated vs. poorly differentiated vs. undifferentiated; (B) N0 stage vs. N1 stage; (C) M0 stage vs. M1 stage; (D) No surgery vs. local excision vs. radical resection; Table S1: Univariate Cox analysis of OS and CSS in the training set.
