# Supplementary material for: Development and Validation of Prognostic Nomograms for Periampullary Neuroendocrine Neoplasms: A SEER Database Analysis
Source: Curr Oncol. 2022 Dec 26;30(1):344–57. doi: 10.3390/curroncol30010028 (PMC9858183; doi:10.3390/curroncol30010028)
Supplement: Supplementary file 1 [file curroncol-30-00028-s001.zip › curroncol-1965226-supplementary.pdf]

Supplementary Material

1 Supplementary Figures and Tables

1.1 Supplementary Figures

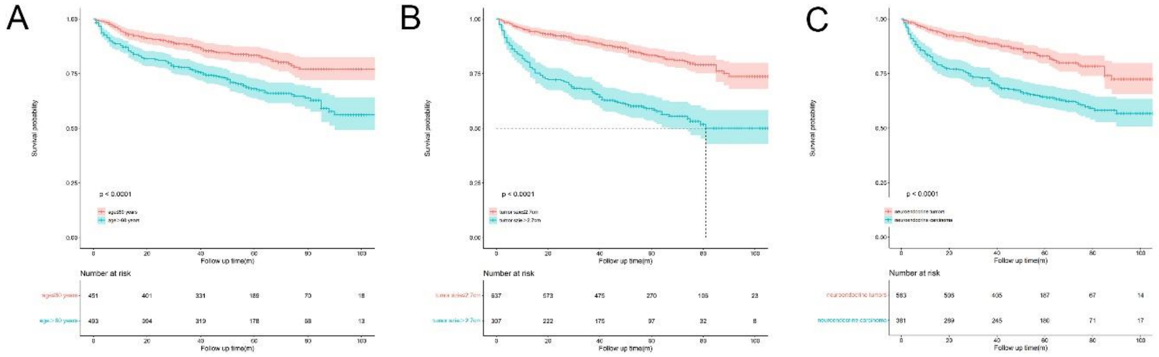

Figure S1. Overall survival analysis in the training set. (A) age ≤ 60 years vs. age >60 years (B) tumor size≤ 2.7cm vs. tumor size> 2.7cm (C) neuroendocrine tumors vs. neuroendocrine carcinoma.

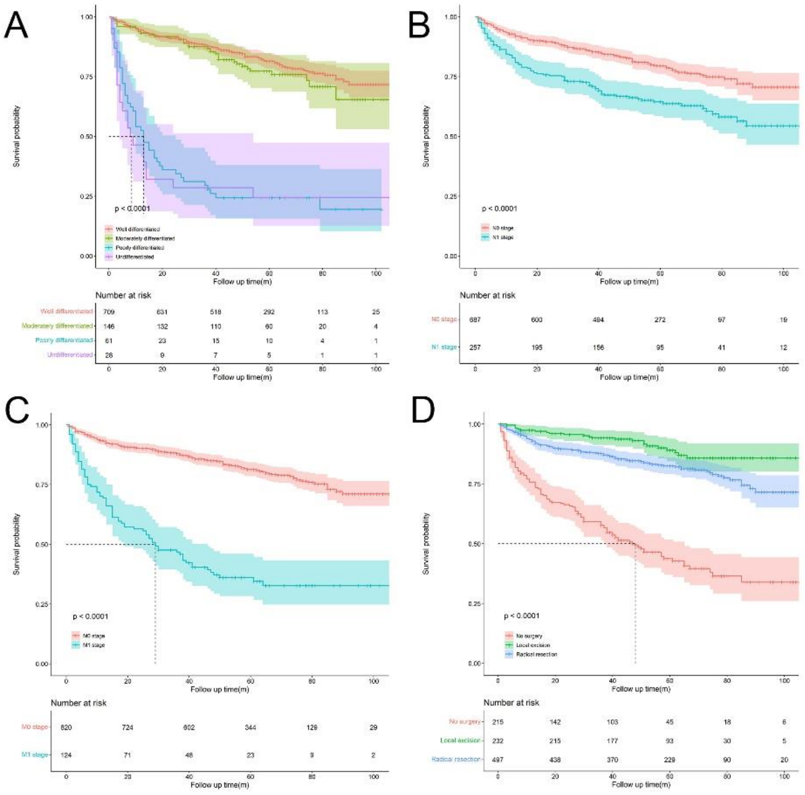

Figure S2. Overall survival analysis in the training set. (A) well differentiated vs. moderately differentiated vs. poorly differentiated vs. undifferentiated; (B) N0 stage vs. N1 stage; (C) M0 stage vs. M1 stage; (D) No surgery vs. local excision vs. radical resection.

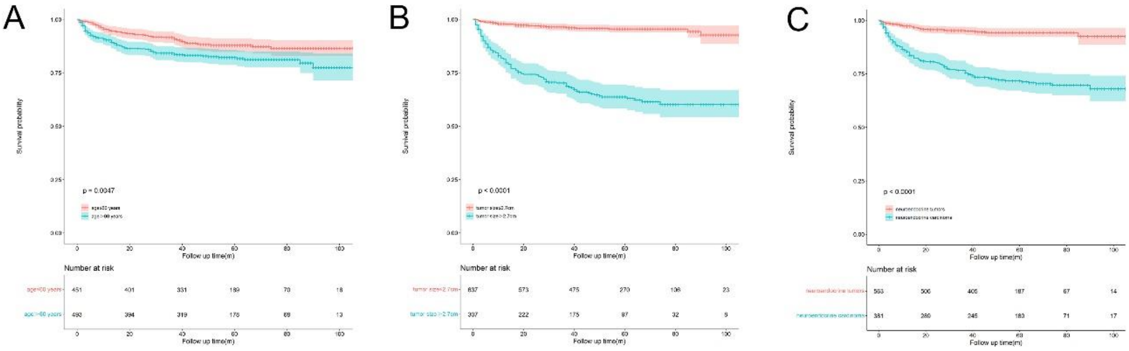

Figure S3. Cancer-specific survival analysis in the training set. (A) age ≤ 60 years vs. age >60 years (B) tumor size≤ 2.7cm vs. tumor size> 2.7cm (C) neuroendocrine tumors vs. neuroendocrine carcinoma.

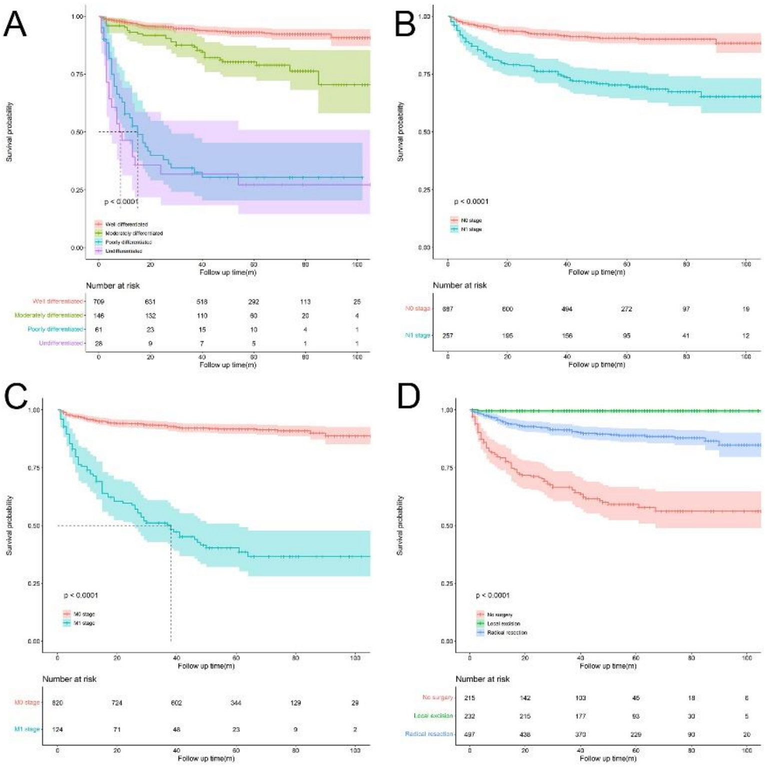

Figure S4. Cancer-specific survival analysis in the training set. (A) well differentiated vs. moderately differentiated vs. poorly differentiated vs. undifferentiated; (B) N0 stage vs. N1 stage; (C) M0 stage vs. M1 stage; (D) No surgery vs. local excision vs. radical resection.

1.2 Supplementary Tables

Table S1 Univariate Cox analysis of OS and CSS in the training set

Variables	HR (95% CI) (OS)	P value	Variables	HR (95% CI) (CSS)	P value
Age			Age		
≤ 60 years	-	-	≤ 60 years	-	-
>60 years	1.99(1.52-2.61)	<0.001	>60 years	1.64(1.16-2.31)	0.005
Sex			Sex		
Female	-	-	Female	-	-
Male	1.03(0.80-1.33)	0.812	Male	0.84(0.60-1.37)	0.295
Race			Race		
White	-	-	White	-	-
Black	1.18(0.87-1.62)	0.289	Black	0.87(0.56-1.37)	0.556
Other	0.73(0.44-1.2)	0.215	Other	0.83(0.46-1.52)	0.552
Geographic region			Geographic region		
Rural/urban	-	-	Rural/urban	-	-
Metropolitan	0.91(0.61-1.35)	0.627	Metropolitan	0.93(0.55-1.56)	0.781
Income ($)			Income ($)		
≤ 60,000	-	-	≤ 60,000	-	-
60,000-70,000	0.86(0.63-1.16)	0.319	60,000-70,000	1.02(0.68-1.53)	0.928
>70,000	0.79(0.58-1.08)	0.143	>70,000	0.98(0.65-1.47)	0.912
Primary site			Primary site		
Duodenum	-	-	Duodenum	-	-
Ampulla	1.66(0.91-3.03)	<0.001	Ampulla	4.19(1.88-9.33)	<0.001
Pancreatic head	1.75(1.34-2.29)	<0.001	Pancreatic head	5.36(3.44-8.36)	<0.001
Tumor size			Tumor size		
≤ 2.7cm	-	-	≤ 2.7cm	-	-
> 2.7cm	3.04(2.35-3.93)	<0.001	> 2.7cm	9.57(6.32-14.49)	<0.001
Histology			Histology		
Neuroendocrine tumors	-	-	Neuroendocrine tumors	-	-
Neuroendocrine carcinomas	2.35(1.81-3.05)	<0.001	Neuroendocrine carcinomas	5.51(3.69-8.22)	<0.001
Differentiation			Differentiation		
Well differentiated	-	-	Well differentiated	-	-
Moderately differentiated	1.24 (0.85-1.81)	0.258	Moderately differentiated	3.04(1.93-4.80)	<0.001
Poorly differentiated	7.64(5.46-10.69)	<0.001	Poorly differentiated	16.73(10.95-25.57)	<0.001
Undifferentiated	8.2(5.17-13.02)	<0.001	Undifferentiated	20.59(12.17-34.83)	<0.001
T stage			T stage		

T1	-	-	T1	-	-
T2	1.63(1.15-2.30)	0.006	T2	5.53(2.74-11.14)	<0.001
T3	2.77(1.99-3.86)	<0.001	T3	14.09(7.24-27.42)	<0.001
T4	5.10(3.28-7.93)	<0.001	T4	27.44(13.27-56.73)	<0.001
N stage			N stage		
N0	-	-	N0	-	-
N1	1.97(1.51-2.55)	<0.001	N1	3.58(2.56-5.00)	<0.001
M stage			M stage		
M0	-	-	M0	-	-
M1	5.00(3.8-6.56)	<0.001	M1	9.71(6.94-13.59)	<0.001
Surgery			Surgery		
No surgery	-	-	No surgery	-	-
Local excision	0.13(0.08-0.20)	<0.001	Local excision	0.01(<0.001-0.06)	<0.001
Radical resection	0.25(0.19-0.32)	<0.001	Radical resection	0.23(0.16-0.32)	<0.001
Lymph node examination			Lymph node examination		
No	-	-	No	-	-
Yes	0.65(0.50-0.84)	0.001	Yes	0.80(0.57-1.12)	

Abbreviation: TNM stage: Tumor-Node-Metastasis stage.
